# Opening the Discussion on the Costs of Pedophilic Disorder as a Mental Health Concern

**DOI:** 10.1007/s10508-025-03406-w

**Published:** 2026-03-14

**Authors:** Till Amelung, Robert J. B. Lehmann, Alexander F. Schmidt, Armin Hoyer, Klaus M. Beier

**Affiliations:** 1 Corporate Member of Freie Universität Berlin and Humboldt-Universität zu Berlin, Institut für Sexualwissenschaft und Sexualmedizin, Charité – Universitätsmedizin Berlin, Luisenstraße 57, 10117 Berlin, Germany; 2 Department of Psychology, Medical School Berlin, Hochschule für Gesundheit und Medizin, Berlin, Germany; 3 Institute of Psychology, Social & Legal Psychology, Johannes Gutenberg Universität Mainz, Mainz, Germany

**Keywords:** Pedophilia, Hebephilia, Child sexual abuse, Distress, Cost of illness, DSM-5-TR

## Abstract

Despite recent advances, pedophilic disorder remains a highly stigmatized and underexplored condition. While its classification as a mental disorder is debated, we adopt this assumption provisionally to apply a cost-of-illness (COI) framework. COI studies, though methodologically limited, offer a pragmatic tool to translate individual impairment into societal costs—thus enabling health policy discussions, identifying service gaps, and supporting early intervention. This article outlines the potential COI associated with pedophilic disorder, distinguishing between cases with offense-related behaviors (child sexual abuse and exploitation material use) and non-offending individuals. We argue that neglecting this condition economically and institutionally has led to avoidable human suffering and may increase long-term societal costs. Applying a COI perspective allows for a more empathetic and preventive public health approach—one that fosters help-seeking, reduces stigma, and ultimately helps prevent sexual victimization of children. By quantifying the hidden burden of pedophilic disorder, COI studies may support more rational, humane, and effective responses to a complex condition. They offer a dual benefit: improving care for affected individuals and reducing harm to others. In doing so, they contribute to a public health framework that neither ignores nor criminalizes, but seeks to prevent.

## Introduction

Pedophilia is characterized by a persistent sexual attraction to prepubescent children. According to current diagnostic manuals, it qualifies as a mental health condition (pedophilic disorder) when it leads to significant distress or impairment or involves sexual behaviors with children. The use of child sexual exploitation and abuse material (CSEAM), while not sufficient on its own to establish a diagnosis, may provide important evidence of pedophilic interests and contribute to the assessment of diagnostic criteria (American Psychiatric Association, 2022). Hebephilia, the sexual attraction to pubescent children, is more prevalent and shares similar clinical and forensic challenges (Stephens et al., 2017) but (after a contested debate) has not been included as a categorical diagnosis in DSM-5 (Singy, 2015).

Pedophilic interest, not necessarily in the extent of a mental disorder, is a statistically rare sexual interest in community populations (Joyal et al., 2015). In a large representative Czech sample (*n* > 5.000 men and women, each), 2.6% of men reported pedophilic sexual fantasies, 3.8% would engage in child sexual abuse (CSA) if it were legal, and 3.1% reported the use of CSEAM in the last six months (Bártová et al., 2021). In women, these numbers accumulated to 0.4%, 0.4%, and 1.5%, respectively. Dombert et al. (2016) found an upper bound of 5.5% of German men (*N* > 8000) indicating any form of adult lifetime sexual interest in prepubescent children (based on sexual behavior or fantasy), with 4.1% reporting respective sexual fantasies, 3.1% potentially criminally relevant sexual behaviors, and 1.7% CSEAM use. Pedophilic preferences indicating stronger self-reported sexual fantasies focusing on prepubescent children than on adults, however, were much more rarely indicated by the male participants (0.1%) surveyed in Dombert et al. (2016).

Although in current classification systems, pedophilic disorder is thus conceptualized as a mental disorder (American Psychiatric Association, 2022; World Health Organization, 1993, 2020), there is a broader debate regarding the classification of sexual interests in general (Heinz, 2015; Joyal, 2021) and pedophilia specifically as mental health conditions (Green, 2002; Malón, 2011). Concerning pedophilia, it has been argued that empirical data are missing to support this psychopathological classification in terms of the impact on individual health and well-being over and above the potential for harm to others associated with the condition (Münch et al., 2020).

In this article, we do not aim to take a definitive stance on whether pedophilic disorder should ultimately be classified as a mental health condition. Instead, we provisionally adopt the current diagnostic classification for the purpose of applying the cost-of-illness (COI) framework. We argue that this approach allows for a systematic investigation of the broader societal and healthcare-related implications of the condition. By translating individual burden into economic terms, COI analyses can inform the debate on classification, while also identifying gaps in service provision and opportunities to improve care and public health responses.

Given the stigma (e.g., Jahnke, 2018 for an overview) and potential legal implications surrounding pedophilic disorder, we aim to show that public health frameworks have failed to generate systematic data on its economic impact. We argue that a COI framework—while methodologically limited (Byford et al., 2000; Onukwugha et al., 2016)—offers a preliminary opportunity to break this cycle: it can provide a rational foundation for policy discussion, funding prioritization, and healthcare system engagement (Hodgson, 1989). Crucially, such framing may also enable a more humanistic and more empathetic societal response—one typically reserved for other (mental) health conditions—by shifting the discourse from stigma and fear to care and prevention.

We further argue that such a shift is not only in the interest of affected individuals, but also of potential victims. By acknowledging and addressing pedophilic disorder as a mental health condition rather than ignoring or criminalizing it in silence, we argue that society gains a realistic chance to prevent one of the condition’s most harmful possible manifestations: the sexual victimization of children. We thus aim to show that a COI framework may serve as both a compass for compassion and a tool for prevention, reducing individual suffering and societal harm alike.

### The Role of Cost-of-Illness Studies in Public Health

The COI framework provides a standardized tool to quantify the economic impact of diseases and health conditions on society. It typically distinguishes between direct costs, indirect costs, and intangible costs (Jo, 2014). Direct costs refer to the expenses directly attributable to the treatment and management of the disease/disorder, such as medical care, hospital stays, medication, and other healthcare services (e.g., psychotherapy). Indirect costs account for the loss of productivity due to illness, disability, or premature death, and can extend to the economic burden placed on caregivers. Intangible costs, although harder to quantify, encompass the reduced quality of life, emotional distress, and pain and suffering experienced by patients and their families (see Table 1).

**Table 1:** Overview of potential costs of illness in pedophilic disorder

Component	Definition/Purpose	Typical operationalization	Example outputs in pedophilic disorder
1. Prevalence /Incidence	Quantifies how many individuals are affected in a given period	Epidemiological data (point, period, lifetime prevalence; annual incidence)	2.6% of men report pedophilic sexual fantasies
			Annual incidence of the disorder (i.e., including distress or behavioral manifestation) unknown
2. Direct medical costs	Costs directly related to diagnosis, treatment, and medical care	Hospital stays, outpatient treatment, medication, rehabilitation	Medical procedures after CSA
			(Ineffective?) care for co-morbid mental disorders in individuals with pedophilia
3. Direct non-medical costs	Costs arising from non-medical services caused by the condition	Social services, transportation, caregiving, supportive programs	Social services for survivors of CSA
			Rehabilitation services for convicted offenders
4. Indirect costs	Productivity losses due to morbidity or premature mortality	Human capital approach, friction cost method	Lost productivity in individuals with pedophilic disorders, e.g., avoiding working with children or due to comorbidity
			Lifetime earnings loss through mental disorders after child sexual victimization
5. Intangible costs	Non-monetary burden on quality of life and well-being	Quality of life adjusted life years (QALYs), Disability adjusted life years (DALYs), willingness-to-pay estimates	QALY reduction due to fear of discovery
			QALY reduction due to giving up having children
			DALY reduction due to suicidality in both survivors and perpetrators of CSA
6. Time horizon	Specifies period over which costs are calculated	Annual, lifetime, multi-year horizon	Pedophilia as a chronic condition versus pedophilic disorder as a potentially curable condition
7. Data sources	Type and quality of input data for costing	Administrative claims, surveys, registries, expert estimates	Dunkelfeld research
			Government data on offender management costs

Example outputs are intended to be illustrative rather than exhaustive. Further examples are given in the text

CSA, Child sexual abuse

COI studies play an important role in informing health policies by identifying the economic consequences of disease. They support decisions about resource allocation, guide interventions, and help evaluate the cost-effectiveness of treatment options (Murray, 2022). To date, COI studies have been conducted for a wide range of both common (e.g., asthma, dementia, substance abuse) and less common (e.g., Prader-Willi syndrome, cystic fibrosis, schizophrenia) conditions (Akobundu et al., 2006; Angelis et al., 2015).

Despite its clinical classification and measurable prevalence, pedophilic disorder has so far been excluded from this field of economic analysis. This gap is particularly striking when compared to conditions of comparable prevalence, such as delusions (Heilskov et al., 2020). The reasons for this neglect are likely diverse. For example, they can be sought in structural stigma, lack of specialized services as well as treatment guidelines or high-quality evidence on their effectiveness, and legal and ethical ramifications—all of which contribute obstacles to data collection and the development of prevention-oriented services.

This article aims to narrow that gap by exploring the potential COI associated with pedophilic disorder. To this end, this article aims to provide a discussion of the potential COI associated with pedophilic disorder, thereby highlighting the neglect of the extensive economic burden it imposes on healthcare systems. In the following, we differentiate between individuals with pedophilic disorder who have engaged in offending behavior (PD–O) and individuals with pedophilic disorder without any offense-behavioral manifestation (PD–NO) or so-called non-offending pedophiles (Cantor & McPhail, 2016) and outline the respective economic dimensions.^1^

### Costs of Illness of Pedophilic Disorder Involving Child Sexual Abuse and Child Sexual Exploitation and Abuse Material Use

Even though pedophilic disorder can manifest through CSA and CSEAM offenses, only about half of convicted offenders of CSA and a slightly larger proportion in CSEAM offenders meet its diagnostic criteria (Seto, 2018). It is thus crucial not to conflate the categorical diagnosis of a pedophilic disorder with the criminal acts of CSA and CSEAM offenses. Estimating associated costs is further complicated by the fact that the majority of both offenses remain unreported and thus never manifest in official statistics of crime. For example, 80% of individuals who sought treatment in the Dunkelfeld project due to their pedophilic interest reported to have used CSEAM, out of which only a minority (16%) had been detected at any time before presentation (Von Franqué et al., 2023). Also, about 36% of individuals who experienced CSA victimization refrained from disclosure even in forensic interviews (Azzopardi et al., 2019), and 12–52% of victims did not disclose experienced sexual abuse until being approached for research purposes (Manay & Collin-Vézina, 2021). Recognizing these specificities is essential to understanding the potential economic burden of pedophilic disorder manifesting through related CSA and CSEAM offenses.

That noted, the primary reason for the availability of COI data for PD–O is the involvement of the legal and criminal justice systems. Resulting costs then include both costs to victims and costs associated with PD–O. It is important to recognize that CSEAM constitutes the graphic conservation of actual acts of CSA. The burden on those depicted in such imagery thus consists of the repercussions brought about by their initial victimization and may be increased due to the ongoing loss of control over the spreading of and access to the documentation of their sexually abusive experiences, exacerbating further both tangible and intangible costs (Gewirtz-Meydan et al., 2018).

Typically, COI studies face inherent methodological challenges (Byford et al., 2000). These include difficulties in attributing costs specifically to one condition when comorbidities exist, challenges in quantifying intangible costs, and the risk of either over- or underestimating economic burden depending on included cost categories. For pedophilic disorder specifically, additional challenges arise from: (1) the hidden nature of the population, making representative sampling difficult; (2) the diagnostic overlap with criminal behavior, complicating the distinction between costs attributable to the disorder versus the offense; (3) the heterogeneity of the population, including those with and without hebephilic interests, varying levels of distress, and different behavioral manifestations. Despite these limitations, COI frameworks provide a structured approach to begin understanding the economic dimensions of this condition.

### Costs of Victimization

The estimated lifetime economic burden for survivors of non-fatal CSA cases that occurred in 2015 in the USA totaled $9.3 billion, with the average cost per survivor ranging from $74,691 to $282,734 (Letourneau et al., 2018). Victimization by PD–O may incur extensive costs, categorizable into direct, indirect, and intangible expenses as well as immediate and long-term costs.

Immediate direct costs incurred by victimization include costs for immediate medical care for physical injuries and acute mental health support for the victims and their care givers. Child welfare costs, such as expenses related to child protective services, foster care, and other professional interventions, form an additional part of the immediate direct costs. Children who develop emotional or behavioral problems following their experience of CSA may go on to require special education services like case management or smaller classes, which further add to the direct costs (Letourneau et al., 2018). Longer-term direct costs include healthcare costs for both mental and physical disorders related to child sexual victimization (Hailes et al., 2019; Irish et al., 2010). Estimating these long-term costs is especially challenging given that many survivors of child sexual abuse cannot or do not want to disclose their victimization history (McElvaney, 2015). Immediate indirect costs include loss of productivity for care givers of survivors of child sexual abuse. Longer-term indirect costs may incur through decreased productivity, increased absenteeism, and loss of educational and career opportunities due to ongoing psychological distress of survivors of CSA as well as increased reliance on social services.

On top, the intangible costs for victims may be significant, encompassing emotional distress, social consequences, and stigmatization. The emotional and psychological sequelae from CSA can lead to conditions such as PTSD, depression, and anxiety (Hailes et al., 2019). These impacts are profound and lasting, affecting the victims’ social relationships and societal perception, leading to isolation and difficulties in social interactions. The reduction in quality of life due to CSA can be measured in terms of quality-adjusted life year (QALY) losses, with the estimated costs increasing significantly when these factors are included (Peterson et al., 2018). The high intangible costs associated with emotional and psychological impairment were highlighted in studies on the economic burden of child maltreatment in the USA and the costs of sexual violence in Iowa (Yang et al., 2014).

### Costs of Managing Offending Individuals with Pedophilic Disorder

Sexual offending behavior against children leads to direct interaction with law enforcement, judicial processes, and incarceration. These interactions generate measurable costs that are well-documented due to the necessity of budget allocations for policing, prosecution, court trials, and imprisonment of (alleged) offenders. Legal systems maintain detailed records and statistics on these processes, which are often analyzed to assess the financial impact on the state and society. Therefore, data on the costs of management of offending individuals are more readily available, encompassing direct, indirect, and intangible costs. Specifically, the annual cost of incarcerating offenders convicted of CSA and CSEAM in the USA is estimated at $5.4 billion (Letourneau et al., 2023). This figure excludes costs incurred prior to incarceration, such as detection and prosecution, or post-release costs like supervision or registration. In addition, costs for child protection agencies are not represented in these figures. Judicial and administrative costs, including trials, appeals, and other legal processes, further add to the direct financial burden.

No monetary estimates exist of the additional costs through broken vocational careers and stress on the families of PD–O (e.g., Armitage et al., 2024; McLaren, 2013). Families may face placement of children in foster care. PD–O often face reduced work capacity or unemployment due to criminal records and incarceration as well as other social adversities such as homelessness and difficulties with social reintegration or family disruption (Kurtz, 2025). This leads to significant productivity losses affecting the overall economy (Letourneau et al., 2018). Rehabilitation programs, aimed at reducing recidivism, also contribute to indirect costs, encompassing both tangible and intangible expenses (Donato & Shanahan, 2001). Long-term healthcare needs, including mental health services, further add to the indirect costs for PD–O (Letourneau et al., 2018).

Intangible costs include emotional distress and stigmatization. The societal and emotional impact of child sexual abuse, including the stigma and psychological trauma experienced by PD–O with pedophilic interests, significantly affects their reintegration into society (Donato & Shanahan, 2001). Community costs, such as fear and reduced sense of safety due to the presence of PD–O, also represent substantial intangible costs (Peterson et al., 2018).

As most CSA cases remain outside the judicial context, i.e., within Dunkelfeld populations (see above; beginning of this subsection), estimates for PD–O and victims are thought to underestimate the actual costs. And while CSA and CSEAM are associated with pedophilic disorder, the available data lack specificity for the condition. The actual costs of pedophilic disorder manifesting through CSA and CSEAM offenses thus remain unknown though even reduced estimations based on the abovementioned numbers may involve considerable economic damage.

### Cost of Illness of Pedophilic Disorder Without Sexual Offending

PD-NO may experience significant internal conflict (e.g., struggling to reconcile their tabooed attractions with their desire to adhere to societal norms; McPhail, 2024) and emotional distress (e.g., fear of discovery Jahnke et al., 2015a, 2015b, 2015c), hopelessness, or low self-worth and suicidal ideation (Ingram et al., 2024, 2025). Disclosure of their condition may lead to social ostracism and legal scrutiny, exacerbating their psychological distress (Elchuk et al., 2022; Jahnke et al., 2024). This population has demonstrably high rates of comorbid conditions such as anxiety, depression, suicidal ideation, and substance use disorders (Gerwinn et al., 2018; for a review, see also Lawrence & Willis, 2021). Although the causes for this increased mental health burden are not well understood, models of pedophilia implying developmental disorders lend to an interpretation in terms of a common etiological root for both pedophilic interests and increased mental health challenges (Fazio, 2018; Fazio et al., 2014). Also, early adverse events are more common in this population than in controls (Gerwinn et al., 2018; Jahnke et al., 2022; Jespersen et al., 2009), which may contribute to this increased mental health burden. A third explanation lies in the pervasive social stigma surrounding pedophilia (Lehmann et al., 2021) that may result in internalized stigma (Jahnke, 2018; Lievesley et al., 2020). For example, depressive symptoms may result from struggling to resolve the conflict between pedophilic desires and the societal rejection of those desires, leading to profound feelings of guilt, hopelessness, and despair (Lawrence & Willis, 2021). Over time, these may escalate into depressive disorders requiring therapeutic intervention. Access to such treatment, however, is often limited for individuals with pedophilic interest due to considerable reluctance among medical and psychological therapists to offer care (e.g., Schmidt & Niehaus, 2022). Similarly, the constant fear of being discovered coupled with the stress of living with a stigmatized condition can manifest as generalized anxiety or social anxiety which may exacerbate to manifest disorders. Also, substance use disorders can be understood as dysfunctional coping strategies to numb aversive emotions or rejected sexual thoughts. Such self-medication can evolve into addiction, creating another layer of complexity in their mental health.

In summary, the increased comorbidities may be conceptualized as resulting from processes that resemble those laid out in the minority stress model for sexual minorities (Meyer, 2003). What’s more, non-offending individuals with pedophilic sexual interest lack a relevant source to cope with this stress as they may face great difficulties finding a community of others experiencing the same challenges in contemporary societies. And while causal evidence for this latter explanation, i.e., the development of comorbidities because of minority stress, is missing, if it were true, any therapeutic aid for the comorbidities not addressing the actual cause is deemed to fail. Indeed, qualitative work with individuals with pedophilic sexual interests from the community has shown a frequent mismatch between available mental health services and the needs of the population (Grady et al., 2019). In addition, the stigmatization creates a barrier to early intervention. Individuals with pedophilic sexual interests often fear being labeled as dangerous, “ticking time bomb” or “sex offenders in waiting” (Jahnke et al., 2024), leading them to hide their condition. As a result, they may avoid seeking help until their condition or comorbidities cause significant psychological distress, thereby exacerbating both.

Either way, comprehensive information on the COI for PD–NO is missing. Research has focused more on prevalence, psychological impact, and treatment, with limited data on the economic burden or healthcare costs for PD–NO. This lack of data highlights yet another critical gap in understanding the broader societal and healthcare implications of the condition.

Based on the preceding argument, the COI associated with pedophilic disorder without any offense-related behavioral manifestation can be separated into costs attributable to the condition itself and costs attributable to comorbid conditions. For example, direct costs may include costs from both specialized medical and psychological services such as diagnostic evaluation, therapy, medication, and unspecific mental health services aimed at managing comorbid disorders such as psychiatric or psychological treatment or addiction medicine services. Indirect costs may result from reduced work performance or unemployment due to comorbid disorders, as well as reduced work capacity among family members or caregivers stemming from their support of the affected individual. Clinical experience suggests additional costs, e.g., through abandoned careers in child-related professions or self-chosen isolation and following unemployment to avoid exposure to children in public places (Heid, 2021).

Intangible costs include the reduced quality of life following the social stigma experienced by PD–NO (Jahnke et al., 2015a, b, c). The emotional and psychological distress associated with the condition can also lead to significant challenges in social relationships and societal perception, resulting in isolation and marginalization. More specific intangible costs may arise from problems in establishing fulfilling intimate relationships with adult partners (Mundy et al., 2022).

### Barriers to Data Collection and the Role of Stigma

The stigma surrounding pedophilia poses a significant challenge to the collection of accurate COI data from both PD–NO and PD–O outside the judicial context. Fear of legal consequences and social rejection may deter individuals from seeking medical help or from participating in research (Grady et al., 2019). For example, in many jurisdictions, individuals seeking help for their pedophilic disorder may risk the involvement of child protective agencies even in the absence of any illegal behavior when clinicians have a justifiable suspicion of a child being at risk of significant harm, e.g., under mandatory reporting statutes in North America and Australia or risk-based child protection frameworks in the UK or Germany (see Iffland & Schmidt, 2023 on the problems of predicting parent–child sexual victimization based on paraphilic sexual interests; Johnston, 2010). This results in underreporting and a lack of comprehensive studies on the condition. Consequently, healthcare systems fall short in recognizing the need for specialized services or the benefits of early intervention and treatment. For example, statistics on hospital treatment rates in Germany show an age-standardized case rate of pedophilic disorder per 100,000 inhabitants of 0 for the years 2000–2022 (www.gbe-bund.de). The absolute registered case numbers range between 10 and 55 per year. These figures not only contrast sharply with the prevalence in empirical research (see above), illustrating the factual barriers for representation in public health statistics. They are also at odds with actual figures of the Dunkelfeld prevention network at approximately 20,000 anonymous contacts in 20 years. Such programs providing anonymous support and therapy have repeatedly shown that individuals with pedophilic disorder will seek help if they are guaranteed confidentiality (Beier et al., 2009, 2015, 2024; Engel et al., 2018; Seto et al., 2025; Von Franqué et al., 2023). This situation is unlike in other mental health conditions such as schizophrenia, where official health statistics allow for a better estimate of the economic impact.

Societal reluctance to recognize pedophilic disorder as a mental health condition that warrants medical and psychological support further limits research. Psychotherapists hesitate to provide support for individuals with pedophilia due to concerns about stigma, legal issues, lack of training in addressing pedophilia, and personal discomfort, further limiting the availability of treatment and hampering research efforts (Jahnke et al., 2015a, b, c; Lievesley et al., 2022; Schmidt & Niehaus, 2022). Focusing solely on crime-related manifestations of pedophilic disorder not only overlooks a substantial proportion of affected individuals but also obscures the heterogeneity of developmental trajectories associated with the condition. While some individuals with pedophilic interests may go on to engage in offending behavior, many do not. Early access to support and intervention may therefore reduce the overall COI by addressing mental health needs, mitigating comorbidities, and, where relevant, reducing situational risk factors associated with potential offending. Failure to provide such support may increase direct, indirect, and intangible costs borne by affected individuals, victims, and their respective families.

### Potential Benefits and Call for Action

The COI associated with pedophilic disorder is diverse and may accumulate to substantial amounts (see Table 1). Acknowledging the COI of pedophilic disorder with or without offence-behavioral manifestations is a crucial first step in improving care for this condition. On the one hand, consistent with its conceptualization as a mental disorder, framing pedophilic disorder as a public health and mental health issue rather than a criminal phenomenon could help to reduce stigma and encourage more people to seek help. Such encouragement may help advance further research to better clarify the support needs for this population (Beier et al., 2024). On the other hand, the absence of comprehensive COI data for PD-NO poses significant policy challenges. Compared to other health conditions for which COI studies have informed policy and funding decisions, the absence of such data for pedophilic disorder points to a notable gap in public health planning, as this condition has not been addressed through explicitly formulated strategies beyond broad, non-specific mental health approaches. In contrast, the lack of comparable data for pedophilic disorder directly constrains the development, prioritization, and evaluation of effective public health responses and support mechanisms for affected individuals.

Without a well-founded understanding of the economic burden of pedophilic disorder, it is difficult to justify spending of public health resources on this mental disorder and to gauge the effectiveness of resource allocation for prevention, treatment, and support services. This gap can lead to inadequate funding, thus hindering the development of therapeutic interventions that help individuals effectively manage their mental health condition (without primarily focusing on risk of offending). COI studies addressing this gap, in contrast, could provide measures to gauge the effectiveness and efficiency of therapeutic and support services, including confidential counseling and tailored treatment programs, to better address the mental health challenges of this population.

COI data can help to inform public education campaigns aimed at reducing stigma. By presenting the economic and social costs of ignoring the needs of PD–NO, these campaigns can shift public perception from viewing pedophilia solely as a criminal issue to recognizing it as a public health concern. Such a shift in perspective may foster a more compassionate and informed dialogue about the disorder, encouraging individuals to seek help earlier and reducing the societal stigma that often surrounds the condition. For individuals with sexual interests in children who (not yet) experience any distress about it and have never committed any related offenses, i.e., individuals with pedophilia but not the associated disorder, such evidence-based de-stigmatization might even bring about opportunities to prevent the development of the disorder altogether.

Finally, comprehensive COI data might be essential for guiding future research and innovation in the treatment and management of pedophilic disorder. By identifying gaps in current knowledge and highlighting areas where new treatments or interventions could be most impactful, COI data can stimulate innovation in the field, ultimately advancing the care for PD–O and PD–NO alike—treatment domains for which, yet, empirically supported therapeutic treatments are missing.

Understanding and adequately addressing the population’s needs and impairments could thus help increase the mental health and well-being of affected individuals as well as reduce the risk of harmful behaviors for themselves and others, thereby ultimately reducing the societal costs associated with pedophilia-related CSA and CSEAM (Letourneau et al., 2018).

This effort must be undertaken while acknowledging the methodological challenges involved, the complexity of factors influencing help-seeking behavior, and the need for COI research to be integrated within a broader framework of prevention science, treatment effectiveness research, and child protection efforts (Beier et al., 2025).

## Limitations

Several caveats need to be borne in mind in this discussion. First and foremost, the COI framework, albeit popular, is not the only health economic tool to inform policy makers. Moreover, the method has seen specific criticism. For example, its results only give an estimate of the costs brought about by any health condition but not of the effectiveness of investments in their mitigation (Onukwugha et al., 2016). This cost-effectiveness depends on additional factors like the effectiveness and efficiency of interventions. COI studies also lack information on the relative value of the affected goods (Currie et al., 2000). As an example, the authors provide comparative cost figures in a ratio of 4:1 for heart disease and traffic injuries, respectively. As the meaning of this ratio is difficult to assess (are heart diseases four times worse than traffic injuries?), the authors question the meaningfulness of any such cost estimates. Furthermore, estimates of indirect costs based on productivity losses implicitly assume that affected individuals would otherwise participate in economic activity (Rice, 1994), an assumption that is particularly problematic for children and elderly persons.

We argue, however, that in the absence of any reliable measure for the COI of pedophilic disorder, such health economic details are of lower priority. Before we are even able to estimate the cost-effectiveness of interventions, an estimate of the costs to be prevented is needed. Such measure is absent thus far for pedophilic disorder with or without prior sexual offending, preventing economically well-informed policy decisions. Concerning the treatment costs in the Dunkelfeld programs financed by health insurance funds, these account for approximately 5–10% of the costs for imprisonment or placement in a forensic hospital while reports of any such incidents, i.e., incarceration of participants in one of the Dunkelfeld projects, have not yet been published.

Strategies to address both the difficulty to assess the relative value placed in illness-related events and the inability of the affected children to partake in the national value chain include adopting a willingness-to-pay methodology, i.e., gauging estimates on the amount of money individuals were willing to spend to prevent an illness-related outcome (Rice, 1994). Again, such studies are lacking for pedophilic disorder, but could provide a valuable addition to other COI studies.

Our analysis focuses specifically on pedophilic disorder as a mental health condition, leaving aside other causes of sexual offenses against minors, which nevertheless may cause significant harm. This focus should not be taken to imply that the costs of these offenses are negligible or should otherwise be subordinate to the ones discussed in this manuscript. Arguments for other conditions associated with sexual offenses against minors paralleling the ones brought forth here can surely be made but would have different implications and should thus be part of a separate discussion. 

Our analysis adopts a mental health framework for pedophilic disorder. This leaves unresolved the tension between clinical and criminal criteria for the disorder (Münch et al., 2020). Adopting a mental health framework for the present argument by no means implies any personal stance on this issue. On the contrary, we believe that gathering COI data under the premise of accepting pedophilic disorder as a mental health condition might provide useful by yielding empirical arguments to this debate about the boundaries and implications of diagnostic systems in stigmatized mental health conditions such as, e.g., the paraphilic disorders.

## Conclusion

Acknowledging COI can help support public health decisions by highlighting the societal costs of a health condition and potential economic benefits of prevention and appropriate care (Akobundu et al., 2006; Angelis et al., 2015; Hodgson, 1989). Concerning pedophilic disorder, such acknowledgment is lacking. Research has addressed the economic costs of the legal response to CSA or CSEAM offenses and the burden of their consequences to victims. In this, an important differentiation for PD–O and PD–NO is lacking. Also, public framing of pedophilic disorder primarily as a criminal rather than a health issue has prevented affected individuals from coming forward and seeking help from mental health professionals. This has led to an under exploration of other associated economic costs, e.g., incurred for PD–O or PD–NO.

We argue that examining the COI of pedophilic disorder with or without offence-behavioral manifestations is imperative. If we uncover the hidden costs of its manifestations with or without offense-related behaviors, we will better be able to gauge the societal response to this condition, including health care and stigma management. Thus, acknowledging the COI of pedophilic disorder thereby is not merely a technical exercise in health economics. Embedding the management of pedophilic disorder into a health economic framework can lay the groundwork for a more humane, effective, and forward-looking public health strategy by helping us to understand the extended value of prevention services—recognizing suffering where it exists and reducing it not only as part of child protection efforts but also as a matter of preventive health care for affected individuals. Crucially, this approach does not merely benefit individuals with pedophilic disorder. By informing a more empathetic and needs-based societal response, COI data may also support broader harm-reduction strategies that aim to reduce the risk of offense-related behaviors and child victimization (Beier et al., 2024). By identifying and addressing mental health needs early and lowering the barriers to seeking help, societal responses may shift toward earlier support and risk-aware intervention, with potential implications for offense-related behavioral outcomes. In doing so, cost-of-illness studies may ultimately serve a dual purpose by laying the groundwork for improved care for individuals with pedophilic disorder while also informing broader harm-reduction efforts, with implications for both human suffering and long-term societal costs associated with child sexual abuse and exploitation.

## Data Availability

Not applicable.

## Footnotes

1 The present article focuses on pedophilic disorder as a diagnosable mental disorder. The substantial costs associated with child sexual abuse are not limited to offences committed by individuals with pedophilic disorder but may incur through acts by individuals with sexual interests in pubescents as well as individuals without any diagnosable sexual interests in minors. These costs are not considered negligible. Rather, we restrict our analysis to pedophilic disorder for reasons of etiological specificity and health-economic attribution.

## References

[CR1] Akobundu, E., Ju, J., Blatt, L., & Mullins, C. D. (2006). Cost-of-illness studies: A review of current methods. *PharmacoEconomics,**24*(9), 869–890. 10.2165/00019053-200624090-00005 16942122 10.2165/00019053-200624090-00005

[CR2] American Psychiatric Association. (2022). *Diagnostic and statistical manual of mental disorders* (Fifth ed., text rev.). American Psychiatric Publishing

[CR3] Angelis, A., Tordrup, D., & Kanavos, P. (2015). Socio-economic burden of rare diseases: A systematic review of cost of illness evidence. *Health Policy,**119*(7), 964–979. 10.1016/j.healthpol.2014.12.016 25661982 10.1016/j.healthpol.2014.12.016

[CR4] Armitage, R., Wager, N., Wibberley, D., Hudspith, L. F., & Gall, V. (2024). We’re not allowed to have experienced trauma. We’re not allowed to go through the grieving process exploring the indirect harms associated with Child Sexual Abuse Material (CSAM) offending and its impacts on non-offending family members. *Victims & Offenders,**19*(5), 915–941. 10.1080/15564886.2023.2172504

[CR5] Azzopardi, C., Eirich, R., Rash, C. L., MacDonald, S., & Madigan, S. (2019). A meta-analysis of the prevalence of child sexual abuse disclosure in forensic settings. *Child Abuse & Neglect,**93*, 291–304. 10.1016/j.chiabu.2018.11.020 30579645 10.1016/j.chiabu.2018.11.020

[CR6] Bártová, K., Androvičová, R., Krejčová, L., Weiss, P., & Klapilová, K. (2021). The prevalence of paraphilic interests in the Czech population: Preference, arousal, the use of pornography, fantasy, and behavior. *Journal of Sex Research,**58*(1), 86–96. 10.1080/00224499.2019.1707468 31916860 10.1080/00224499.2019.1707468

[CR7] Beier, K. M., Ahlers, C. J., Goecker, D., Neutze, J., Mundt, I. A., Hupp, E., & Schaefer, G. A. (2009). Can pedophiles be reached for primary prevention of child sexual abuse? First results of the Berlin Prevention Project Dunkelfeld (PPD). *Journal of Forensic Psychiatry & Psychology,**20*(6), 851–867. 10.1080/14789940903174188

[CR8] Beier, K. M., Grundmann, D., Kuhle, L. F., Scherner, G., Konrad, A., & Amelung, T. (2015). The German Dunkelfeld Project: A pilot study to prevent child sexual abuse and the use of child abusive images. *Journal of Sexual Medicine,**12*(2), 529–542. 10.1111/jsm.12785 25471337 10.1111/jsm.12785

[CR9] Beier, K. M., Nentzl, J., Von Heyden, M., Fishere, M., & Amelung, T. (2024). Preventing child sexual abuse and the use of child sexual abuse materials: Following up on the German Prevention Project Dunkelfeld. *Journal of Prevention,**45*, 881–890. 10.1007/s10935-024-00792-0 39269516 10.1007/s10935-024-00792-0 PMC11568044

[CR10] Beier, K. M., Von Heyden, M., Nentzl, J., & Amelung, T. (2025). Preventing child sexual abuse in the ‘Dunkelfeld’: A public health imperative requiring context-appropriate science—A response to König (2025) [Letter to the Editor]. *Journal of Prevention,**46*, 785–792. 10.1007/s10935-025-00859-6 40576918 10.1007/s10935-025-00859-6 PMC12553596

[CR11] Byford, S., Torgerson, D. J., & Raftery, J. (2000). Economic note: Cost of illness studies. *BMJ (Clinical Research Ed.),**320*(7245), 1335. 10.1136/bmj.320.7245.1335 10807635 10.1136/bmj.320.7245.1335 PMC1127320

[CR12] Cantor, J. M., & McPhail, I. V. (2016). Non-offending pedophiles. *Current Sexual Health Reports,**8*(3), 121–128. 10.1007/s11930-016-0076-z

[CR13] Currie, G., Kerfoot, K. D., Donaldson, C., & Macarthur, C. (2000). Are cost of injury studies useful? *Injury Prevention,**6*(3), 175–176. 10.1136/ip.6.3.175 11003180 10.1136/ip.6.3.175 PMC1730644

[CR100] Dombert, B., Schmidt, A. F., Banse, R., Briken, P., Hoyer, J., Neutze, J., & Osterheider M. (2016). How common is males’ self-reported sexual interest in prepubescent children? *Journal of Sex Research,**53*(2), 214–223 26241201 10.1080/00224499.2015.1020108

[CR14] Elchuk, D. L., McPhail, I. V., & Olver, M. E. (2022). Stigma-related stress, complex correlates of disclosure, mental health, and loneliness in minor-attracted people. *Stigma and Health,**7*(1), 100–112. 10.1037/sah0000317

[CR15] Engel, J., Körner, M., Schuhmann, P., Krüger, T. H. C., & Hartmann, U. (2018). Reduction of risk factors for pedophilic sexual offending. *Journal of Sexual Medicine,**15*(11), 1629–1637. 10.1016/j.jsxm.2018.09.001 30297101 10.1016/j.jsxm.2018.09.001

[CR16] Fazio, R. L. (2018). Toward a neurodevelopmental understanding of pedophilia. *Journal of Sexual Medicine,**15*(9), 1205–1207. 10.1016/j.jsxm.2018.04.631 29861362 10.1016/j.jsxm.2018.04.631

[CR17] Fazio, R. L., Lykins, A. D., & Cantor, J. M. (2014). Elevated rates of atypical handedness in paedophilia: Theory and implications. *Laterality: Asymmetries of Body, Brain and Cognition,**19*(6), 690–704. 10.1080/1357650X.2014.898648 10.1080/1357650X.2014.898648 PMC4151814 24666135

[CR18] Gerwinn, H., Weiß, S., Tenbergen, G., Amelung, T., Födisch, C., Pohl, A., Massau, C., Mohnke, S., Kärgel, C., Wittfoth, M., Jung, S., Drumkova, K., Walter, M., Beier, K. M., Walter, H., Ponseti, J., Schiffer, B., & Kruger, T. H. C. (2018). Clinical characteristics associated with paedophilia and child sex offending – Differentiating sexual preference from offence status. *European Psychiatry,**51*, 74–85. 10.1016/j.eurpsy.2018.02.002 29625377 10.1016/j.eurpsy.2018.02.002

[CR19] Gewirtz-Meydan, A., Walsh, W., Wolak, J., & Finkelhor, D. (2018). The complex experience of child pornography survivors. *Child Abuse & Neglect,**80*, 238–248. 10.1016/j.chiabu.2018.03.031 29631255 10.1016/j.chiabu.2018.03.031

[CR20] Grady, M., Levenson, J., Mesias, G., Kavanagh, S., & Charles, J. (2019). “I can’t talk about that”: Stigma and fear as barriers to preventive services for minor-attracted persons. *Stigma and Health,**4*, 400–410. 10.1037/sah0000154

[CR21] Green, R. (2002). Is pedophilia a mental disorder? *Archives of Sexual Behavior,**31*(6), 467–471. 10.1023/A:1020699013309 12462476 10.1023/a:1020699013309

[CR22] Hailes, H. P., Yu, R., Danese, A., & Fazel, S. (2019). Long-term outcomes of childhood sexual abuse: An umbrella review. *The Lancet Psychiatry,**6*(10), 830–839. 10.1016/S2215-0366(19)30286-X 31519507 10.1016/S2215-0366(19)30286-X PMC7015702

[CR23] Heid, L. (2021). What can we help you with? A qualitative study of the experiences of non-offending men with a sexual interest in children leading to their seeking professional help. ATSA conference 2021, virtual. https://www.atsa.com/student#collapseSix

[CR24] Heilskov, S. E. R., Urfer-Parnas, A., & Nordgaard, J. (2020). Delusions in the general population: A systematic review with emphasis on methodology. *Schizophrenia Research,**216*, 48–55. 10.1016/j.schres.2019.10.043 31836260 10.1016/j.schres.2019.10.043

[CR25] Heinz, A. (2015). *Der Begriff der psychischen Krankheit* (2. Aufl). Suhrkamp

[CR26] Hodgson, T. A. (1989). Cost of illness studies: No aid to decision making? Comments on the second opinion by Shiell et al. (Health Policy, 8(1987) 317–323). *Health Policy*, *11*(1), 57–60. 10.1016/0168-8510(89)90055-9 10.1016/0168-8510(89)90055-9 10312927

[CR27] Iffland, J. A., & Schmidt, A. F. (2023). Stigmatization and perceived dangerousness for intrafamilial child sexual abuse of fathers with a history of sexual offenses and paraphilic interests: Results from a survey of legal psychological experts. *Child Abuse & Neglect,**144*, 106348. 10.1016/j.chiabu.2023.106348 37478734 10.1016/j.chiabu.2023.106348

[CR28] Ingram, M., Letourneau, E. J., & Nestadt, P. S. (2024). Themes associated with suicidal ideation and behavior among people attracted to children. *Archives of Sexual Behavior,**53*(4), 1343–1360. 10.1007/s10508-023-02770-9 38200329 10.1007/s10508-023-02770-9

[CR29] Ingram, M., Thorne, E., Letourneau, E. J., & Nestadt, P. S. (2025). Self-esteem, perceived social support, and suicidal ideation and behavior among adults attracted to children. *Omega,**91*(3), 1214–1235. 10.1177/00302228221150304 36630479 10.1177/00302228221150304

[CR30] Irish, L., Kobayashi, I., & Delahanty, D. L. (2010). Long-term physical health consequences of childhood sexual abuse: A meta-analytic review. *Journal of Pediatric Psychology,**35*(5), 450–461. 10.1093/jpepsy/jsp118 20022919 10.1093/jpepsy/jsp118 PMC2910944

[CR31] Jahnke, S. (2018). The stigma of pedophilia: Clinical and forensic implications. *European Psychologist,**23*(2), 144–153. 10.1027/1016-9040/a000325

[CR32] Jahnke, S., Blagden, N., McPhail, I. V., & Antfolk, J. (2024). Secret-keeping in therapy by clients who are sexually attracted to children. *Psychotherapy Research,**34*(7), 941–956. 10.1080/10503307.2023.2265047 37848189 10.1080/10503307.2023.2265047

[CR33] Jahnke, S., Imhoff, R., & Hoyer, J. (2015). Stigmatization of people with pedophilia: Two comparative surveys. *Archives of Sexual Behavior,**44*(1), 21–34. 10.1007/s10508-014-0312-4 24948422 10.1007/s10508-014-0312-4

[CR34] Jahnke, S., Philipp, K., & Hoyer, J. (2015). Stigmatizing attitudes towards people with pedophilia and their malleability among psychotherapists in training. *Child Abuse & Neglect,**40*, 93–102. 10.1016/j.chiabu.2014.07.008 25085206 10.1016/j.chiabu.2014.07.008

[CR35] Jahnke, S., Schmidt, A. F., Geradt, M., & Hoyer, J. (2015). Stigma-related stress and its correlates among men with pedophilic sexual interests. *Archives of Sexual Behavior,**44*(8), 2173–2187. 10.1007/s10508-015-0503-7 25933669 10.1007/s10508-015-0503-7

[CR36] Jahnke, S., Schmidt, A. F., Klöckner, A., & Hoyer, J. (2022). Neurodevelopmental differences, pedohebephilia, and sexual offending: Findings from two online surveys. *Archives of Sexual Behavior,**51*(2), 849–866. 10.1007/s10508-021-02228-w 34993718 10.1007/s10508-021-02228-w PMC8888371

[CR37] Jespersen, A. F., Lalumière, M. L., & Seto, M. C. (2009). Sexual abuse history among adult sex offenders and non-sex offenders: A meta-analysis. *Child Abuse & Neglect,**33*(3), 179–192. 10.1016/j.chiabu.2008.07.004 19327831 10.1016/j.chiabu.2008.07.004

[CR38] Jo, C. (2014). Cost-of-illness studies: Concepts, scopes, and methods. *Clinical and Molecular Hepatology,**20*(4), 327–337. 10.3350/cmh.2014.20.4.327 25548737 10.3350/cmh.2014.20.4.327 PMC4278062

[CR39] Johnston, S. (2010). The unintended effect in mandatory reporting laws and an increased risk to a protected population [Editorial]. *Columbia Social Work Review,**8*, 1–4.

[CR40] Joyal, C. C. (2021). Problems and controversies with psychiatric diagnoses of paraphilia. In L. A. Craig (Ed.), *Sexual deviance* (1st ed., pp. 91–116). Wiley. 10.1002/9781119771401.ch6

[CR41] Joyal, C. C., Cossette, A., & Lapierre, V. (2015). What exactly is an unusual sexual fantasy?: Sexual fantasies in the general population. *Journal of Sexual Medicine,**12*(2), 328–340. 10.1111/jsm.12734 25359122 10.1111/jsm.12734

[CR42] Kurtz, D. (2025). *Sex offenders: An overlooked significant subpopulation homeless*. Cicero Institute. ciceroinstitute.org

[CR43] Lawrence, A. L., & Willis, G. M. (2021). Understanding and challenging stigma associated with sexual interest in children: A systematic review. *International Journal of Sexual Health,**33*(2), 144–162. 10.1080/19317611.2020.1865498 38596748 10.1080/19317611.2020.1865498 PMC10906971

[CR44] Lehmann, R. J. B., Schmidt, A. F., & Jahnke, S. (2021). Stigmatization of paraphilias and psychological conditions linked to sexual offending. *Journal of Sex Research,**58*(4), 438–447. 10.1080/00224499.2020.1754748 32352329 10.1080/00224499.2020.1754748

[CR45] Letourneau, E. J., Brown, D. S., Fang, X., Hassan, A., & Mercy, J. A. (2018). The economic burden of child sexual abuse in the United States. *Child Abuse & Neglect,**79*, 413–422. 10.1016/j.chiabu.2018.02.020 29533869 10.1016/j.chiabu.2018.02.020 PMC6542279

[CR46] Letourneau, E. J., Roberts, T. W. M., Malone, L., & Sun, Y. (2023). No check we won’t write: A report on the high cost of sex offender incarceration. *Sexual Abuse,**35*(1), 54–82. 10.1177/10790632221078305 35318871 10.1177/10790632221078305

[CR47] Lievesley, R., Harper, C. A., & Elliott, H. (2020). The internalization of social stigma among minor-attracted persons: Implications for treatment. *Archives of Sexual Behavior,**49*(4), 1291–1304. 10.1007/s10508-019-01569-x 31925747 10.1007/s10508-019-01569-x PMC7145785

[CR48] Lievesley, R., Swaby, H., Harper, C. A., & Woodward, E. (2022). Primary health professionals’ beliefs, experiences, and willingness to treat minor-attracted persons. *Archives of Sexual Behavior,**51*(2), 923–943. 10.1007/s10508-021-02271-7 35084616 10.1007/s10508-021-02271-7 PMC8793822

[CR49] Malón, A. (2012). Pedophilia: A diagnosis in search of a disorder. *Archives of Sexual Behavior,**41*(5), 1083–1097. 22367174 10.1007/s10508-012-9919-5

[CR50] Manay, N., & Collin-Vézina, D. (2021). Recipients of children’s and adolescents’ disclosures of childhood sexual abuse: A systematic review. *Child Abuse & Neglect,**116*, 104192. 10.1016/j.chiabu.2019.104192 31564382 10.1016/j.chiabu.2019.104192

[CR51] McElvaney, R. (2015). Disclosure of child sexual abuse: delays, non-disclosure and partial disclosure. What the research tells us and implications for practice. *Child Abuse Review,**24*(3), 159–169. 10.1002/car.2280

[CR52] McLaren, H. J. (2013). (Un)‐blaming mothers whose partners sexually abuse children: In view of heteronormative myths, pressures and authorities. *Child & Family Social Work,**18*(4), 439–448. 10.1111/j.1365-2206.2012.00863.x

[CR53] McPhail, I. V. (2024). The subjective experience of individuals with pedohebephilic interest. *Current Sexual Health Reports,**16*(1), 35–46. 10.1007/s11930-023-00381-y

[CR54] Meyer, I. H. (2003). Prejudice, social stress, and mental health in lesbian, gay, and bisexual populations: Conceptual issues and research evidence. *Psychological Bulletin,**129*(5), 674–697. 10.1037/0033-2909.129.5.674 12956539 10.1037/0033-2909.129.5.674 PMC2072932

[CR55] Münch, R., Walter, H., & Müller, S. (2020). Should behavior harmful to others be a sufficient criterion of mental disorders? Conceptual problems of the diagnoses of antisocial personality disorder and pedophilic disorder. *Frontiers in Psychiatry,**11*, 558655. 10.3389/fpsyt.2020.558655 33093836 10.3389/fpsyt.2020.558655 PMC7523554

[CR56] Mundy, C. L., Lewis, H. L., & Cioe, J. D. (2022). Romantic and sexual relationships with adult partners among pedohebephilic men. *Archives of Sexual Behavior,**51*(2), 911–921. 10.1007/s10508-021-02108-3 34799829 10.1007/s10508-021-02108-3

[CR57] Murray, C. J. L. (2022). The Global burden of disease study at 30 years. *Nature Medicine,**28*(10), 2019–2026. 10.1038/s41591-022-01990-1 36216939 10.1038/s41591-022-01990-1

[CR58] Onukwugha, E., McRae, J., Kravetz, A., Varga, S., Khairnar, R., & Mullins, C. D. (2016). Cost-of-illness studies: An updated review of current methods. *PharmacoEconomics,**34*(1), 43–58. 10.1007/s40273-015-0325-4 26385101 10.1007/s40273-015-0325-4

[CR101] Peterson, C., Florence, C., & Klevens, J., (2018). The Economic Burden of Child Maltreatment in the United States, 2015. *Child Abuse & Neglect,**86*, 178–183. 10.1016/j.chiabu.2018.09.018 30308348 10.1016/j.chiabu.2018.09.018 PMC6289633

[CR59] Rice, D. P. (1994). Cost of illness—Fact or fiction? *The Lancet,**344*(8936), 1519–1520. 10.1016/S0140-6736(94)90343-3 10.1016/s0140-6736(94)90342-5 7983947

[CR60] Schmidt, A. F., & Niehaus, S. (2022). Outpatient therapists’ perspectives on working with persons who are sexually interested in minors. *Archives of Sexual Behavior,**51*(8), 4157–4178. 10.1007/s10508-022-02377-6 35939157 10.1007/s10508-022-02377-6 PMC9663344

[CR61] Seto, M. C. (2018). *Pedophilia and sexual offending against children: Theory, assessment, and intervention* (2018–33719–005). American Psychological Association. 10.1037/0000107-005

[CR62] Seto, M. C., Roche, K., Coleman, J., Findlater, D., & Letourneau, E. J. (2025). Self-help seeking for people concerned about their thoughts and behaviors regarding children during the COVID-19 pandemic. *Sexual Offending: Theory, Research, and Prevention,**20*, Article e14301. 10.5964/sotrap.14301 41716198 10.5964/sotrap.14301 PMC12914599

[CR106] Shanahan, M., & R. Donato. (2001). Counting the cost: Estimating the economic benefit of pedophile treatment programs. *Child Abuse & Neglect**25*(4), 541–555. http://www.ncbi.nlm.nih.gov/pubmed/11370725 11370725 10.1016/s0145-2134(01)00225-3

[CR63] Singy, P. (2015). Hebephilia: A postmortem dissection. *Archives of Sexual Behavior,**44*(5), 1109–1116. 10.1007/s10508-015-0542-0 25894647 10.1007/s10508-015-0542-0

[CR64] Stephens, S., Seto, M. C., Goodwill, A. M., & Cantor, J. M. (2017). Evidence of construct validity in the assessment of hebephilia. *Archives of Sexual Behavior,**46*(1), 301–309. 10.1007/s10508-016-0907-z 27900492 10.1007/s10508-016-0907-z

[CR65] Von Franqué, F., Bergner-Koether, R., Schmidt, S., Pellowski, J. S., Peters, J. H., Hajak, G., & Briken, P. (2023). Individuals under voluntary treatment with sexual interest in minors: What risk do they pose? *Frontiers in Psychiatry,**14*, 1277225. 10.3389/fpsyt.2023.1277225 38076694 10.3389/fpsyt.2023.1277225 PMC10701425

[CR66] Author. (1993). *The ICD-10 classification of mental and behavioural disorders*. World Health Organization.

[CR67] Author. (2020). *ICD-11 for mortality and morbidity statistics*. World Health Organization. https://icd.who.int/

[CR103] Yang, J., Miller, T. R., Zhang, N., LeHew, B., & Peek-Asa C. (2014). Incidence and Cost of Sexual Violence in Iowa. *American Journal of Preventive Medicine,**47*(2), 198–202. 10.1016/j.amepre.2014.04.005 24930620 10.1016/j.amepre.2014.04.005

